# Probing the stress and depression circuits with a disease gene

**DOI:** 10.7554/eLife.10829

**Published:** 2015-09-15

**Authors:** Chang Sin Park, X William Yang

**Affiliations:** Center for Neurobehavioral Genetics, Jane and Terry Semel Institute for Neuroscience and Human Behavior and the Department of Psychiatry and Biobehavioral Sciences, David Geffen School of Medicine, University of California, Los Angeles, Los Angeles, United Statesparkcs@ucla.edu; Center for Neurobehavioral Genetics, Jane and Terry Semel Institute for Neuroscience and Human Behavior and the Department of Psychiatry and Biobehavioral Sciences, David Geffen School of Medicine, University of California, Los Angeles, Los Angeles, United Statesxwyang@mednet.ucla.edu

**Keywords:** depression, stress, anxiety, medial prefrontal cortex, wolfram syndrome, neuropsychiatric disorders, mouse

## Abstract

Selectively deleting a gene that has been linked to depression from specific neurons in mice sheds new light on a neural circuit that controls stress-induced depressive behaviors.

**Related research article** Shrestha P, Mousa A, Heintz N. 2015. Layer 2/3 pyramidal cells in the medial prefrontal cortex moderate stress induced depressive behaviors. *eLife* **4**:e08752. doi: 10.7554/eLife.08752

**Image** If a protein called WFS1 (red) is not produced in particular neurons, stressed mice are more likely to behave in ‘depression-like’ ways

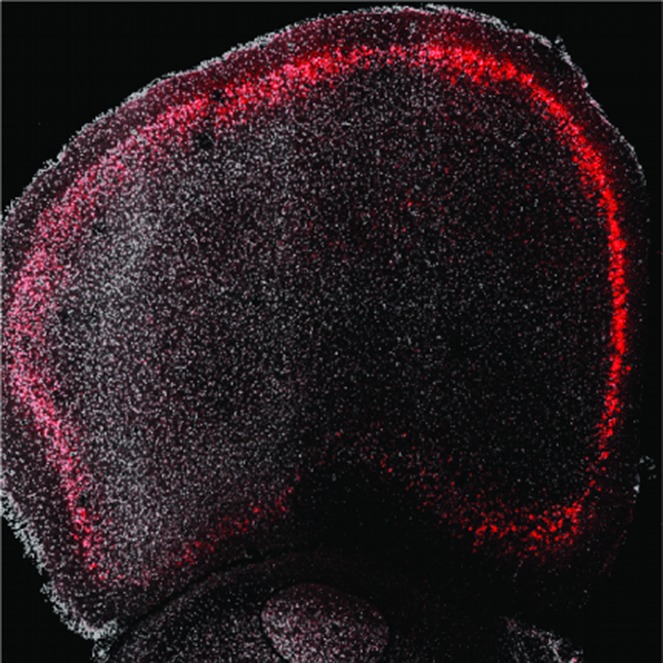

Depression afflicts about 350 million people globally. It is the leading cause of disability worldwide and one of the top causes of death in adults. The first treatment for depression was discovered more than 50 years ago, but current antidepressants improve symptoms in less than half of patients. The major hurdle to discovering new therapies for depression is the lack of a true biological understanding of the disorder. Its causes are likely to be complex, and may involve numerous genetic and environmental risk factors (Flint and Kendler, 2014). Now, in eLife, Prerana Shrestha, Awni Mousa and Nathaniel Heintz at the Rockefeller University reveal more about the role of a specific group of neurons in depressive behaviors (Shrestha et al., 2015).

Human studies suggest that depression involves multiple brain regions. This is not unexpected, given the range of symptoms linked to the disease: from depressed or agitated moods and a decreased interest in pleasurable activities, to insomnia or hypersomnia, changes in weight and suicidal ideas or actions. However, it is known that experimental deep brain stimulation of a specific cortical area can reduce the symptoms of depression in treatment-resistant patients (Mayberg et al., 2005). This underscores the importance of studying how individual neural circuits contribute to the disorder.

Although rodents are not expected to exhibit the full spectrum of depression symptoms, environmental stresses, which are known risk factors for depression in humans, can cause the animals to act in ways that are considered ‘depression-like’ (Nestler and Hyman, 2010). Recent advances in neurotechnologies have provided tremendous insights into the ways that different kinds of neurons and neural circuits influence whether the rodents are susceptible or resilient to stress-induced depressive behaviors (Deisseroth, 2014). However, there are gaps in our knowledge of what links particular neurons and underlying molecular mechanisms to depression.

An alternative approach, which has been used in studying Mendelian disorders such as Huntington's disease (Wang et al., 2014), is to dissect the pathological neural circuit with the disease gene itself. Shrestha et al. now provide an elegant example of applying neurogenetic modeling in mice to study the neural circuits linked to depression. They studied the gene *WFS1*, which is mutated in a disorder called Wolfram syndrome. Patients with this syndrome suffer from diabetes, progressive damage to the optic nerve, deafness, and an array of neurological and psychiatric symptoms including anxiety and depression (Rigoli et al., 2011). Both copies of *WFS1* must be mutated to cause Wolfram syndrome. Intriguingly, carriers of the disease who only have one mutant copy of the gene show core symptoms of depression (without displaying other symptoms of Wolfram syndrome) and are more likely to be admitted to psychiatric hospitals (Swift and Swift, 2005).

Consistent with a role for *WFS1* in mood disorders, the gene is expressed at high levels in brain regions implicated in emotion and reward processing (Takeda et al., 2001). A previous study of mice that lacked the *Wfs1* gene showed some symptoms consistent with Wolfram syndrome, but depressive behaviors were not consistently observed (Luuk et al., 2009).

Shrestha et al. found that *Wfs1* is selectively expressed in a specific population of neurons (the layer 2/3 pyramidal neurons) in the prefrontal cortex of mice. Given that the medial prefrontal cortex (mPFC) has been linked to depression and behavioral resilience (e.g., Russo and Nestler, 2013), could *Wfs1* in mPFC neurons regulate stress-induced depressive behaviors?

To address this question, Shrestha et al. developed a mouse model that allows the part of the *Wfs1* gene that is commonly mutated in people with Wolfram syndrome to be deleted in selected cells. They showed that “conditional knockout” (CKO) mice with *Wfs1* deleted from all the pyramidal neurons of the cortex and hippocampus generally act normally. However, when these CKO mice are acutely restrained, they show multiple depressive behaviors, as well as heightened stress hormone release. When *Wsf1* was deleted only from pyramidal neurons in the mPFC, the mice still displayed stress-induced depressive behaviors. The Wfs1 protein in mPFC layer 2/3 neurons therefore appears to be crucial for regulating stress-induced depressive behaviors (Figure 1).

**Figure 1.**
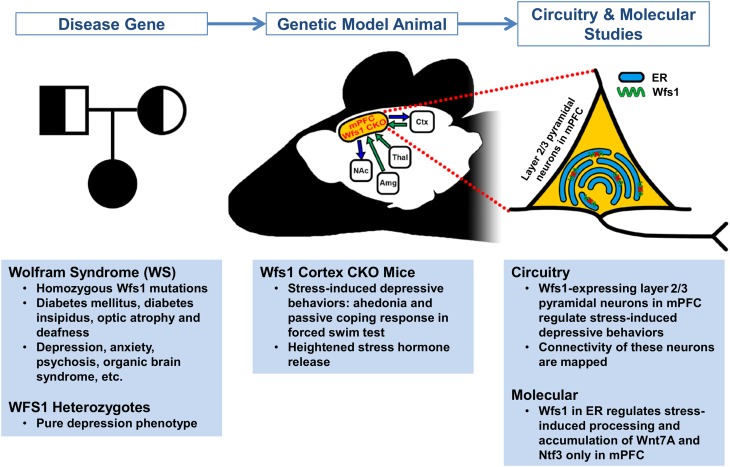
Using a mouse model to determine the molecular mechanisms and neural circuits behind depressive behaviors. People with two mutated copies of Wolfram syndrome gene 1 (*WFS1*) develop a disorder characterized by a wide range of symptoms, whereas people with only one mutant version of this gene are more likely to develop depression. To explore whether this gene controls depressive behaviors through the medial prefrontal cortical (mPFC) circuit – a region previously implicated in stress, depression, and behavioral resilience – Shrestha et al. studied the *Wfs1* gene in mice. First they found that the expression of *Wfs1* was enriched in layer 2/3 pyramidal neurons of the mPFC. Next they deleted *Wfs1* in the cortex and the mPFC to generate Wfs1 cortex CKO mice, which exhibit stress-induced depressive behaviors and elevated stress hormone release. Shrestha et al. also investigated how the mPFC neurons connect to other brain regions (such as the nucleus accumbens (NAc), amygdala (Amg), thalamus (Thal) and cortex (Ctx)), and the effects of *Wfs1* on endoplasmic reticulum (ER) function. Taken together, these results reveal details about the molecular pathways of *Wfs1*-expressing mPFC neurons that regulate depressive behaviors. This also provides a starting point for future studies to narrow down pathogenic mechanisms and identify potential novel therapeutic targets.

Using a disease gene to model pathological behaviors in animals makes it possible to discover critical neural circuits and to study the underlying molecular pathways that control such behaviors. Shrestha et al. have made inroads into both areas, in the first case by using tracing methods to define how *Wfs1*-expressing layer 2/3 neurons in the mPFC connect with other brain regions. Since the Wfs1 protein is found in the endoplasmic reticulum membrane, Shrestha et al. also examined whether the function of this organelle is altered in the neurons of CKO mice. Changes to how the endoplasmic reticulum works were only seen in mPFC neurons, and only when the mice were stressed. Exactly how this influences the behavior of CKO mice still needs clarifying, as does the mechanism by which environmental stress signals are detected and processed by the mPFC neurons.

Our biological understanding of complex psychiatric disorders, such as depression, is about to reach a tipping point. Recent rapid advances in genetic technologies and large-scale patient sample collections have accelerated the discovery of the genes that increase the risk of common psychiatric disorders such as schizophrenia (Schizophrenia Working Group of the Psychiatric Genomics Consortium, 2014). A recent study of 5303 Chinese women with severe, recurrent depression has yielded the first two genome locations associated with the disease (CONVERGE consortium, 2015). It is expected that hundreds more such genetic variants may be discovered (Flint and Kendler, 2014), with each contributing relatively small effects to the overall risk of depression.

The study by Shrestha et al. showcases the prowess of using model animals that carry disease gene variants to dissect the neural circuits that underpin depressive behaviors. In the cases that such models also capture phenotypic aspects of depression, they could transform the study of disease circuits and molecular mechanisms. This may help researchers to discover new therapies for depression, which are five decades overdue.
